# End-of-Life Practices in an Intensive Care Unit of a Private Hospital in Mexico

**DOI:** 10.1089/pmr.2024.0023

**Published:** 2024-08-21

**Authors:** Roberto Carlos Miranda-Ackerman, Paulina Ruiz-Ochoa, Daniela López-Ramírez, Juan Fernando Quevedo-Barrientos, Mariana Plascencia-Rendón, José Luis Landeros-Torres, Karen Fernanda Astorga-Cervantes, Alejandra González-Uribe, Ana Olivia Cortes-Flores, Carlos José Zuloaga-Fernández- del-Valle, Gilberto Morgan-Villela, Francisco José Barbosa-Camacho, Clotilde Fuentes-Orozco, Irma Valeria Brancaccio-Pérez, Alejandro González-Ojeda

**Affiliations:** ^1^ Hospital San Javier, Guadalajara, México.; ^2^ Department of Psychiatry, Hospital Civil de Guadalajara “Fray Antonio Alcalde”, University of Guadalajara, Guadalajara, México.; ^3^ Biomedical Research Unit 02, Hospital de Especialidades, Centro Médico Nacional de Occidente. Guadalajara, Jalisco, México.; ^4^ Department of Anesthesiology, Hospital Civil Juan I. Menchaca, Guadalajara, Mexico.; ^5^ Faculty of Medicine, Universidad de Colima, Colima, México.

**Keywords:** End-of-life practices, critical care medicine, ICU, withdrawal treatment, withholding treatment, cardiopulmonary resuscitation, active shortening of the dying process.

## Abstract

**Background:**

Many factors, such as religion, geography, and customs, influence end-of-life practices. This variability exists even between different physicians.

**Objective:**

To observe and describe the end-of-life actions of patients in the intensive care unit (ICU) and document the variables that might influence decision-making at the end of life.

**Materials and Methods:**

This is a cross-sectional study performed in the ICU patients of a private hospital from March 2017 to March 2022. We used the Philips Tasy Electronic Medical Record database of clinical records; 298 patients were included in the study during these five years (2017–2022). The data analysis was done with the statistical package SPSS version 23 for Windows.

**Results:**

A total of 297 patients were included in this study, of which more than half were men. About 60% of our sample had private health insurance, whereas the remaining paid out of pocket. Most patients had withholding treatment, followed by failed cardiopulmonary resuscitation, withdrawal treatment, and brain death, and none of the patients had acceleration of the dying process. The main cause of admission to the ICU in our center was respiratory complications. Most of our samples were Catholics.

**Conclusions:**

Decision-making at the end of life is a complex process. Active participation of the patient, when possible, the patient’s family, doctors, and nurses, can give different perspectives and a more compassionate and individualized approach to end-of-life care.

## Introduction

Worldwide, 16–23% of patients admitted to an intensive care unit (ICU) died during hospitalization. There are five main categories at the end of life: withdrawal treatment, withhold treatment, failed cardiopulmonary resuscitation (CPR), brain death, and active shortening of the dying process^1^ (pp. 1101–1110). Variability exists from one country to another and even between ICUs; multiple factors are involved in the decision-making at the end of life^2^ (pp. 111–121).

There is not a universal definition for withdrawal and withholding of treatment. However, limitations of life-sustaining interventions can include intravenous fluids, nutrition, cardiovascular support (vasopressors, CPR), and ventilatory support.

Over the years, medical practice has evolved to share decision-making between patients, families, and doctors. Patients’ expectations and preferences have become a priority when making medical decisions, especially at the end of life. Considering the patients’ wishes is a strategy to avoid extending their suffering by not having complex and invasive treatments that will not improve the outcome^3–5^ (pp. 790–797, 439–445, 44–49).

Decision-making at the end of life is influenced by the culture and customs of the country of origin, family involvement, religion, doctors’ beliefs, and ICU practices, among others^4^ (pp. 439–445). A holistic approach is required, mostly because it improves patient care in the ICU at the end of life^2^ (pp. 111–121).

During the study ETHICUS-2, Mexico was included as a part of North America; even though geographically it belongs to this region, cultural differences are notable. In this study, we believe actions at the end of life will have more similarities to Latin American countries included in the ETHICUS-2 study^1^ (pp. 1101–1110).

In 2020, according to the National Institute of Statistics and Geography (INEGI), 78.4% of the Mexican population were Catholics. Despite Mexico’s official status as a secular state, it stands as the second-largest country in terms of Catholic population. We believe religion plays an important role in decision-making at the end of life; in this case, we think Catholic beliefs will influence our results^6^ (p. 684).

The objective of this study was to observe and describe the actions at the end of life for patients in the ICU and to document the variables that might influence decision-making at the end of life.

## Materials and Methods

### Design

This is a cross-sectional study performed in the ICU patients of a private hospital in Mexico from March 2017 to March 2022.

### Participants

During these five years (2017–2022), we obtained a population of 1904 patients in the ICU, of which 297 patients were included in this study. It took place in a quaternary care private-teaching hospital in a developing country. The inclusion criteria were patients admitted to the ICU in one center who died or had a limitation on life-sustaining treatment. Patients discharged from the ICU or with missing data in their medical files were excluded.

### Instruments

The Philips Tasy Electronic Medical Record^®^ was used. The study variables were gender, age, health care coverage, comorbidities, discharge status, religion, complication, mortality, ventilatory support, and nutrition therapy.

### Data analysis

The statistical analysis was performed using the statistical package IBM^®^ SPSS Statistics 25th version for Windows. Raw numbers, mean, percentages, and standard deviation were used for the descriptive analysis. The inferential analysis was performed using the chi-squared test for qualitative variables. For quantitative variables, the Student’s *t* test was used. A *p* value of <0.05 was considered statistically significant.

### Ethics declaration

The authors assert that all procedures contributing to this work comply with the ethical standards of the relevant national and institutional committees on human experimentation and with the Helsinki Declaration of 1975, as revised in Fortaleza, Brazil, in 2013. This work was approved by the ethics committee of Hospital San Javier, registered with the identifier 001-06-2022-RMCA.

## Results

During these five years, from March 2017 to February 2022, 297 patients were hospitalized with a critical or terminal disease. The mean age of our sample was 67.11 years old. Most of the patients were men (62.6%, *n* = 186), and 37.4% (*n* = 111) were women. The 64.6% (*n* = 192) of our sample had private health insurance, and the remaining 35.4% (*n* = 105) paid out of pocket. In general, the average hospital stay was 21.18 ± 58.43 days. The average hospital stay in patients with private health insurance was 25.13 ± 71.70 days, compared with an average of 13.97 ± 13.99 days in those who pay out of pocket; however, there was no significant difference (*p* = 0.116). Catholicism was the predominant religion, with 85.5% (*n* = 254), while 14.5% (*n* = 43) had different religions/ideologies (7.7% (*n* = 23) of patients did not declare any religion, 4% (*n* = 14) did not had any religion, 1.3% (*n* = 4) reported to be Jewish, and 0.7% (*n* = 2) patients reported to be atheists).

There are five main categories at the end of life: withdrawal treatment, withhold treatment, failed CPR, brain death, and active shortening of the dying process (the complete list of the population’s characteristics according to their category can be found in Table 1). In our ICU, withholding treatment was the most prevalent, with 74.4% (*n* = 221), followed by failed CPR with 21.9% (*n* = 65), withdrawal treatment with 2.7% (*n* = 8), and brain death with 1% (*n* = 3). There was no patient who had an acceleration of the death process. When comparing the main categories at the end of life and their religious beliefs, there were no statistical differences between Catholic patients and non-Catholic patients (*p* = 0.464).

**Table 1.** Study Population Characteristics according to Their Categories at the End of Life

	Withholding treatment (*n* = 221)	Withdrawal treatment (*n* = 8)	Failed cardiopulmonary resuscitation (*n* = 65)	Brain death (*n* = 3)	All patients (*n* = 297)
Age (years)	28–96 (ø67)	47–69 (ø69)	23–94 (ø67)	32–89 (ø59)	23–96 (ø67)
Sex					
Male	140 (47.1%)	5 (1.6%)	38 (12.7%)	3 (1%)	186 (62.6%)
Female	81 (27.3%)	3 (1%)	27 (9%)	0 (0%)	111 (37.4%)
ICU admission cause					
Respiratory	110 (37%)	2 (0.6%)	30 (10.1%)	1 (0.3%)	143 (48.1%)
Cardiovascular	31 (10.4%)	2 (0.6%)	14 (4.7%)	0 (0%)	47 (15.8%)
Surgery	18 (6.1%)	0 (0%)	3 (1%)	0 (0%)	21 (7.1%)
Neurological	8 (2.7%)	0 (0%)	6 (2%)	0 (0%)	14 (4.7%)
Sepsis	11 (3.7%)	2 (0.6%)	2 (0.6%)	0 (0%)	15 (5%)
Gastrointestinal	15 (5%)	1 (0.3%)	3 (1%)	0 (0%)	19 (6.4%)
Metabolic	2 (0.6%)	0 (0%)	0 (0%)	0 (0%)	2 (0.6%)
Trauma	1 (0.3%)	0 (0%)	3 (1%)	1 (0.3%)	5 (1.7%)
Cancer	21 (7%)	0 (0%)	4 (1.3%)	1 (0.3%)	26 (8.7%)
Hematological	1 (0.3%)	0 (0%)	0 (0%)	0 (0%)	1 (0.3%)
Miscellaneous	3 (1%)	1 (0.3%)	0 (0%)	0 (0%)	4 (1.3%)
Private health insurance					
Yes	142 (47.8%)	5 (1.7%)	43 (14.5%)	2 (0.7%)	192 (64.6%)
No	79 (26.6%)	3 (1%)	22 (7.4%)	1 (0.3%)	105 (35.3%)
Acute complication					
Respiratory	76 (25.6%)	1 (0.3%)	19 (6.4%)	0 (0%)	96 (32.3%)
Cardiovascular	24 (8%)	1 (0.3%)	15 (5%)	1 (0.3%)	41 (13.4%)
Electrolyte imbalance	2 (0.7%)	0 (0%)	0 (0%)	0 (0%)	2 (0.7%)
Hypovolemic Shock	8 (2.7%)	0 (0%)	6 (2%)	0 (0%)	14 (4.7%)
Metabolic	1 (0.3%)	0 (0%)	1 (0.3%)	0 (0%)	2 (0.7%)
Infection	19 (6.4%)	1 (0.3%)	2 (0.7%)	0 (0%)	22 (7.4%)
Multiple organic dysfunction	88 (29.6%)	4 (1.3%)	21 (7%)	1 (0.3%)	114 (38.4%)
Neurological	3 (1%)	1 (0.3%)	1 (0.3%)	1 (0.3%)	6 (2%)
Religion					
Catholicism	190 (64%)	8 (2.7%)	54 (18.2%)	2 (0.7%)	254 (85.5%)
Undeclared	19 (6.4%)	0 (0%)	3 (1%)	1 (0.3%)	23 (7.7%)
Atheist	0 (0%)	0 (0%)	2 (0.7%)	0 (0%)	2 (0.7%)
Agnostic	9 (3%)	0 (0%)	5 (1.7%)	0 (0%)	14 (4.7%)
Jewish	1 (0.3%)	0 (0%)	1 (0.3%)	0 (0%)	2 (0.7%)
Other	2 (0.7%)	0 (0%)	0 (0%)	0 (0%)	2 (0.7%)

The 77.77% (*n* = 231) of our sample had some comorbidity. Hypertension was the most prevalent comorbidity, affecting 61.47% (*n* = 142) of the patients who had comorbidity, followed by diabetes with 35.93% (*n* = 83), neurological with 11.68% (*n* = 27), and hypothyroidism with 9.52% (*n* = 22). Some patients presented with more than one comorbidity simultaneously.

More than half of the patients had a weight abnormality, and only 33.67% (*n* = 100) had a normal body mass index (BMI) between 18.5 and 24.9 kg/m^2^. The 34% (*n* = 101) of patients were overweight with a BMI between 25 and 29.9 kg/m^2^, 27.94% (*n* = 83) were obese with a BMI above 30 kg/m^2^, and the remaining 4.37% (*n* = 13) were underweight with a BMI below 18.5 kg/m^2^.

We divided the cause of admission to the ICU into 11 categories. Respiratory complications were the main motive for admission with 48.1% (*n* = 143), followed by cardiovascular complications with 15.8% (*n* = 47), cancer complications with 8.8% (*n* = 26), postsurgical with 7.1% (*n* = 21), gastrointestinal complications with 6.4% (*n* = 19), sepsis with 5.1% (*n* = 15), neurological complications with 4.7% (*n* = 14), and others with 4% (*n* = 12). Other causes of admission included trauma, miscellaneous, metabolic, and hematological.

Patients had a critical status during ICU hospitalization; however, they developed an acute complication that led to death. We divided these complications into eight categories. Multiple organic failure was the main complication, found in 38.4% (*n* = 114), followed by acute respiratory failure in 32.3% (*n* = 96), cardiovascular complication in 13.8% (*n* = 41), infectious complication in 7.4% (*n* = 22), hypovolemic shock in 4.7% (*n* = 14), neurological complications in 2% (*n* = 6), electrolyte imbalance in 0.7% (*n* = 2), and metabolic complication in 0.7% (*n* = 2).

Most patients were under some type of ventilatory support, 95.6% (*n* = 284), and only 4.4% (*n* = 13) had no ventilatory support. The 53.52% (*n* = 152) who required ventilatory support were under invasive mechanical ventilation, 30.98% (*n* = 88) had nasal cannula, and 15.14% (*n* = 43) had noninvasive mechanical ventilation (NIMV).

About a third of our sample (31.31%; *n* = 93) was in the ICU due to COVID-19 complications. We found that all patients with COVID required ventilatory support; 75.26% (*n* = 70) had mechanical invasive ventilation, 13.97% (*n* = 13) had NIMV, and only 10.8% (*n* = 10) had nasal cannula. The 44% (*n* = 41) were overweight, 32.3% (*n* = 30) were obese, and only 23.6% (*n* = 22) had a normal weight. There were no underweight patients with COVID-19.

## Discussion

During these five years of study, we aimed to see end-of-life actions in the ICU of a private hospital in Mexico. The “actions” studied were withdrawal treatment, withholding treatment, failed CPR, brain death, and active shortening of the dying process. We believe action is not an adequate term because we cannot include brain death as an action; that is the main reason why, instead of saying actions at the end of life, we decided to say categories at the end of life^2^ (pp. 111–121).

We only included patients who died during hospitalization. Even though there were other patients with terminal disease, if they were discharged from the ICU, it was not possible to keep track of them, so they were excluded. More than half of our sample were men, and the main cause of ICU admission was a respiratory complication.

We compared our results with the ETHICUS-2 study. The mean age of our sample is 68.5 years old; it is very similar to the mean age of the ETHICUS-2 study, which is 66.5 years. This could be explained as elderly patients may more commonly present complications from multiple underlying pathologies. Also, similarly to the ETHICUS-2 cohort, male patients had a higher prevalence than female patients. Our sample’s median length of hospital stay was 21 days, compared with the median of 4 days in ETHICUS-2. However, the authors mention an average hospital stay in Latin America of 3–15 days; although it is more similar to our sample, it is shorter when compared with our study^1^ (pp. 1101–1110). In ETHICUS-2, the most frequent action was withholding treatment, followed by withdrawal treatment, failed CPR, brain death, and a minimal proportion of active shortening of the dying process. The main category in our sample was withholding treatment, but unlike the ETHICUS-2 global cohort and the North American cohort, it was followed by failed CPR, withdrawal treatment, and brain death. It is important to mention that we had no patients with active shortening of the dying process, mainly because it is not legal in our country. It is mentioned in ETHICUS-2 that there was a higher prevalence of failed CPR in Africa and Latin America, and these regions had the lowest rates of withdrawing treatment, which is similar to our results^1^ (pp. 1101–1110). Mexico has more cultural similarities with Latin American countries than North America (North America was the region into which Mexico was grouped together). In the ETHICUS-2 study, death due to failed CPR occurred in 1799 (14%) of the study population, but there was more failed CPR in Africa (106 of 162, 65·4%) and Latin America (160 of 571, 28%); many deaths likely occurred before limitations were implemented and correlated with failed CPR, preceding death in substantially more patients than in other regions. The remaining comparisons can be found in Table 2.

**Table 2.** ETHICUS-2 Sample and This Study Sample Comparison

	ETHICUS-2	ETHICUS-2 (North American cohort)	ICU in private hospital in Mexico
Mean Age	66.5		68.5
Sex	Male 7651 (59.5%) Female 5199 (40.5%)		Male 186 (62.6%) Female 111 (37.4%)
Median length of hospital stay	4 days		21.18 ± 58.43 days
End-of-life practice	Withholding treatment (44.1%) Withdrawal treatment (36.4%) Failed CPR (14%) Brain death (5.1%) Shortening of dying process (0.5%)	Withholding treatment (54%) Withdrawal treatment (36%) Failed CPR (8%) Brain death (1%) Shortening of dying process (0.5%)	Withholding treatment (74.4%) Failed CPR (21.9%) Withdrawal treatment (2.7%) Brain death (1%) Shortening of dying process (0%)

CPR, cardiopulmonary resuscitation; ICU, intensive care unit.

Previous studies have shown that up to 71% of patients in the ICU cannot make decisions on their own, depending on surrogate decision-makers. In the past year’s end of life, decisions have become more and more recognized, especially across European countries. When a patient is in the ICU, it means they are critically ill, and, frequently, they lose autonomy and self-determined decision-making. Due to this loss of independence, having a written advance directive helps preserve a patient’s will and preferences and define who will be the surrogate^7–11^ (pp. 699–710, 261–269, 1–10, 311–316). Even though having an advanced directive should be foreseen, considering that ICU patients are critically ill and can worsen at any moment, it is not something that is always done. Some countries, especially European ones such as Germany, use advance directives regulated by laws^11^ (pp. 311–316).

In Mexican laws, specifically in the general law of health, it is written that any adult person in the use of all their faculties, independently of health condition, can express their advanced written will to receive or not receive any treatment in case of being critically ill and unable to manifest their will. Nevertheless, it is very common that patients do not have an advance directive, and in the end, their families make the last call^2^ (pp. 111–121).

It is documented that practices in end-of-life care are highly variable. This variability relies on different factors such as religion, geography, and culture. Even in a region, there exists variability between ICUs and between individual intensivists^12–13^ (pp. 1572–1585). This variability at different levels makes it harder to study this end-of-life care category because the comparison between studies can be biased. However, the research and knowledge of this topic can help create more standardized directives worldwide, improving end-of-life care decision-making^12^ (pp. 1572–1585).

As mentioned before, worldwide variability exists, and the lack of standardized directives makes this topic very subjective. For example, there is not a universal definition for withdrawal and withholding treatment, so these can be interpreted differently; for one, withholding treatment might include not intubating or not doing CPR; for others, it might include not giving intravenous fluids.^13^

In some places, withdrawal and withhold treatments are considered ethically equivalent, yet there exists a considerable difference in how withhold treatment is done much more than withdrawal treatment. This might be because withdrawal is an active process requiring a written order and justification. In contrast, withholding is a passive process. However, the ideal would be to have written order and justification for what actions are being avoided^14–15^ (pp. 105–140, 1316).

About 85% of our samples were Catholics, which is very similar to our national statistics. This remains relevant to how religion may influence decision-making at the end of life. The Catholic Church, like many other religions, opposes euthanasia, placing a strong emphasis on the sanctity of life^14^ (pp. 105–140.) This is a controversial subject, and it is very hard to measure because we only know the patient’s religion. However, we do not know exactly how they feel about this matter. In most countries, as in ours, it is not a legal procedure, so it is not a viable option^15^ (p. 1316).

## Limitations

It is a one-center study, so our results may differ from other centers. Also, an oversight in our study was the absence of data on the advanced care directives within our sample. This information could have been valuable, as it would have allowed us to explore the relationship between individuals’ choices and key variables such as religion and insurance. However, lacking insight into the precise reasons behind their decisions would have introduced subjectivity into our interpretations.

## Conclusions

Making decisions regarding end-of-life actions in the ICU is a complex and sensitive matter that requires active involvement from patients, their families, and health care professionals. Clinical practice can differ significantly across different contexts, cultures, and countries; there exists variability even between different ICUs.

It is crucial to emphasize that end-of-life practices proved to be highly variable, influenced by different factors such as religion, geography, and customs. We have observed that the decision-making should be collaborative, allowing active participation from physicians, nurses, family members, and, whenever possible, the patient. This multifaceted collaboration acknowledges the diversity of perspectives and values and encourages a more compassionate and personalized approach to end-of-life care.

It is important to understand that there is no right or wrong in decision-making; it should always be individualized. The most important thing to remember is the patient’s comfort, trying to make this process less difficult. As mentioned before, we believe this can be achieved by having the active participation of all involved, always keeping a respectful and empathic environment, and never forgetting that the main goal is to provide dignified end-of-life care.

## Institutional Review Board Statement

The study was conducted in accordance with the Declaration of Helsinki and approved by the Institutional Review Board (or Ethics Committee) of Hospital San Javier registered with the identifier 001-06-2022-RMCA.

## Notes

**Cite this article as:** Miranda-Ackerman RC, Ruiz-Ochoa P, López-Ramírez D, Quevedo-Barrientos JF, Plascencia-Rendón M, Landeros-Torres JL, Astorga-Cervantes KF, González-Uribe A, Cortés-Flores AO, Morgan-Villela G, Barbosa-Camacho FJ, Fuentes-Orozco C, Brancaccio-Pérez IV, and González-Ojeda A. (2024) End-of-life practices in an intensive care unit of a private hospital in Mexico, *Palliative Medicine Reports* 5:1, 359–364, DOI: 10.1089/pmr.2024.0023.
